# Reduced number of cardiovascular events and increased cost-effectiveness by genotype-guided antiplatelet therapy in patients undergoing percutaneous coronary interventions in the Netherlands

**DOI:** 10.1007/s12471-016-0873-z

**Published:** 2016-08-29

**Authors:** B. A. L. M. Deiman, P. A. L. Tonino, K. Kouhestani, C. E. M. Schrover, V. Scharnhorst, L. R. C. Dekker, N. H. J. Pijls

**Affiliations:** 1 Clinical Laboratory, Catharina Hospital, Eindhoven, The Netherlands; 2 Department of Cardiology, Catharina Hospital, Eindhoven, The Netherlands

**Keywords:** Clopidogrel, Prasugrel, CYP2C19, Stent thrombosis, Cost-effectiveness

## Abstract

**Aim:**

This study explores clinical outcome in cytochrome P450 2C19 (CYP2C19)-related poor metaboliser patients treated with either clopidogrel or prasugrel after percutaneous coronary intervention (PCI) and investigates whether this could be cost-effective.

**Methods and results:**

This single-centre, observational study included 3260 patients scheduled for elective PCI between October 2010 and June 2013 and followed for adverse cardiovascular events until October 2014. Post PCI, CYP2C19 poor metaboliser patients were treated with clopidogrel or prasugrel, in addition to aspirin. In total, 32 poor metabolisers were treated with clopidogrel and 41 with prasugrel. The number of adverse cardiovascular events, defined as death from cardiovascular cause, myocardial infarction, stent thrombosis, every second visit to the catheterisation room for re-PCI in the same artery, or stroke, within 1.5 years of PCI, was significantly higher in the CYP2C19 poor metaboliser group treated with clopidogrel (*n* = 10, 31 %) compared with the poor metaboliser group treated with prasugrel (*n* = 2, 5 %) (*p* = 0.003). Costs per gained quality-adjusted life years (QALY) were estimated, showing that genotype-guided post-PCI treatment with prasugrel could be cost-effective with less than € 10,000 per gained QALY.

**Conclusion:**

This study provides evidence that for CYP2C19-related poor metabolisers prasugrel may be more effective than clopidogrel to prevent major adverse cardiovascular events after PCI and this approach could be cost-effective.

## Introduction

Over the last decade, dual antiplatelet therapy with aspirin and clopidogrel has been considered the gold standard therapy for patients undergoing elective percutaneous coronary interventions (PCI) in preventing major adverse cardiovascular events (MACE) [1]. However, resistance to clopidogrel is a well-described phenomenon, with 15–30 % of patients having inadequate platelet inhibition while on therapy [1, 2]. Both clinical factors and genetic polymorphisms are involved in resistance to clopidogrel [2, 3].

Clopidogrel is a pro-drug that requires biotransformation to generate an active metabolite involving cytochrome P450 (CYP) enzymes [4]. It was shown that the CYP2C19 enzyme plays a crucial role in this metabolism [5–7]. Clopidogrel-treated patients carrying at least one reduced-function CYP2C19 allele were shown to have a significantly higher risk for MACE, especially stent thrombosis, than non-carriers [6–9]. These results were confirmed by many studies and two meta-analyses [2, 10, 11]. At the end of 2010 these findings resulted in a Food and Drug Administration (FDA) boxed warning advising healthcare professionals to consider using other antiplatelet medication or alternative dosing strategies for clopidogrel in CYP2C19 poor metaboliser patients.

Therefore, in September 2010 Catharina Hospital started to determine the CYP2C19 genotype of patients scheduled for elective PCI in order to identify CYP2C19 poor metabolisers. At first, poor metabolisers were treated with clopidogrel. After the FDA boxed warning was placed, prasugrel was prescribed instead of clopidogrel to the poor metabolisers, with the goal to reduce the number of MACE. In this paper the results of this therapy change are presented. In addition, the cost-effectiveness of genotype-guided post-PCI treatment is discussed.

## Methods

### Design, setting and definitions

Between September 2010 and June 2013, patients who were scheduled for elective PCI at Catharina Hospital in Eindhoven were enrolled in this study. Patients with ST-segment elevation myocardial infarction (STEMI) who received primary PCI were not included. CYP2C19 genotyping was performed for all patients. Initially, all patients were treated with a start-up dose of clopidogrel (300 mg) and, post-PCI, clopidogrel (75 mg daily) in addition to aspirin (80 mg daily) was prescribed for at least one year. But since the FDA boxed warning, gradually only prasugrel (10 mg daily) instead of clopidogrel was prescribed to CYP2C19 poor metabolisers within 1 to 5 days of PCI, starting in September 2011. Thereby, two groups of CYP2C19 poor metabolisers were studied, one group treated with clopidogrel and another with prasugrel. As stent thrombosis was shown by autopsy to be the cause of death 492 days after PCI for one of the deceased patients in our study, all CYP2C19 poor metabolisers were followed for at least 1.5 years after PCI, the last patient until October 2014. For all CYP2C19 poor metabolisers clinical baseline characteristics were recorded and the medical history was examined for cardiovascular events. An adverse cardiovascular event was defined as death from a cardiovascular cause, myocardial infarction, stent thrombosis, every second visit to the catheterisation room for re-PCI in the same artery, or stroke. Stent thrombosis was defined according to Academic Research Consortium (http://circ.ahajournals.org/content/115/17/2344). For verification of medication prescribed after PCI, the medical file was consulted or the pharmacy or general practitioner was contacted.

### Blood sampling, genotyping and phenotyping

Blood for genotyping was obtained during scheduled blood collection immediately before PCI. Genomic DNA was isolated from the patient’s blood using a commercial DNA extraction kit (Roche Diagnostics Inc.) and a semi-automatic extraction device, the Magnapure (Roche). CYP2C19 genotyping was performed with real-time polymerase chain reaction and melting temperature analysis using the Lightcycler hybridisation DNA master mix (Roche Diagnostics Inc.) and specific primers and probes for detection of the CYP2C19*2 and *3 alleles and the *17 allele (TIP-MOLBIOL and Sigma-proligo) on the Lightcycler 480 (Roche). Based on the genotypes, patients with CYP2C19 wild-type were phenotyped as extensive metabolisers, CYP2C19*2 heterozygote and CYP2C19*3 heterozygote patients as intermediate metabolisers, CYP2C19*2 homozygotes as poor metabolisers and CYP2C19*17 homozygotes as ultra-rapid metabolisers. The CYP2C19*17 heterozygote patients were phenotyped as extensive metabolisers and CYP2C19*2*17 as intermediate metabolisers.

### Data analysis

For the baseline characteristics, variables with a Gaussian distribution were presented as mean ± standard deviation. *P*-values were evaluated by a Chi-squared or Fisher’s exact test using the SPSS statistics software (IBM), version 19 for statistical analyses.

The cost-effectiveness analysis was based on the low-discrimination scenario of Kazi and colleagues[12], including direct medical costs, induced costs, and acute event costs, and in addition to stent thrombosis also cardiovascular death other than stent thrombosis (e. g. bleeding) was included.

## Results

### CYP2C19 allele frequencies

A total of 3260 patients were enrolled in this study of which 2293 were wild-type, 932 were heterozygotes and 89 homozygotes for the CYP2C19*2 allele. In addition, 6 patients were heterozygotes for the CYP2C19*3 allele, 1121 were heterozygotes for the CYP2C19*17 allele and 175 patients were homozygotes for the CYP2C19*17 allele. This results in allele frequencies of 17, 0.1 and 22.5 % for CYP2C19*2, CYP2C19*3 and CYP2C19*17, respectively, which is in agreement with the allele frequency of a normal Caucasian population [13].

### Cardiovascular events

**Fig. 1:**
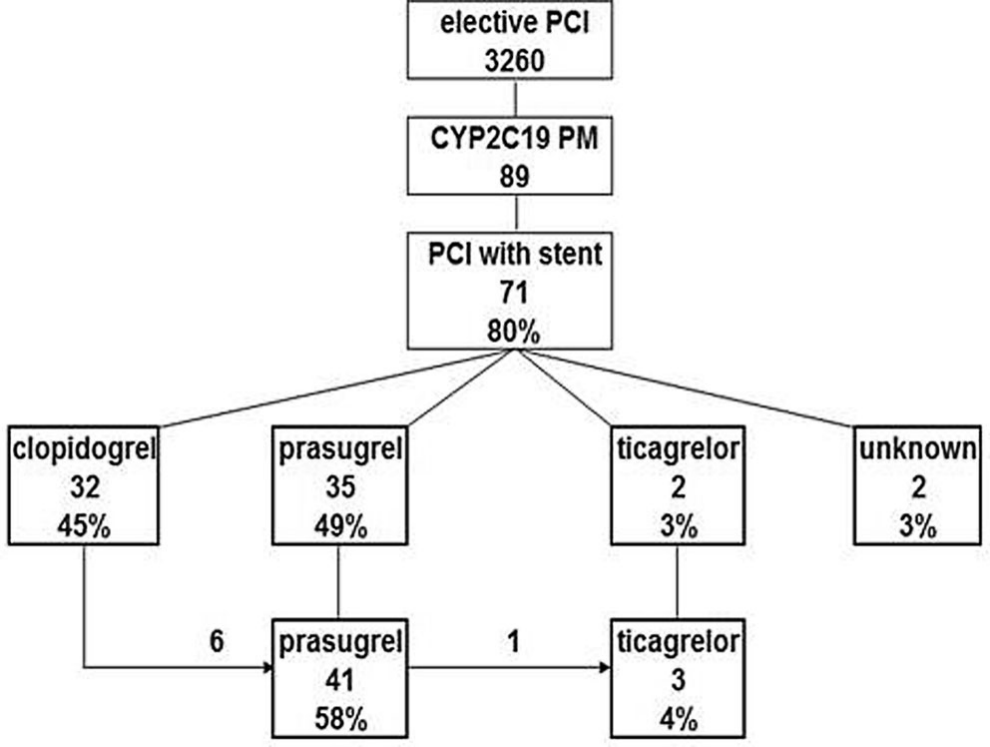
Antiplatelet treatment of CYP2C19 poor metabolisers after PCI with stent placement. The number of patients scheduled for elective PCI, phenotyped as CYP2C19 poor metabolisers, and who received a stent is indicated. Patients were subdivided based on the drug used for antiplatelet therapy. For 6 patient using clopidogrel, prasugrel was prescribed after the event, and for one patient treated with prasugrel, ticagrelor was prescribed after the event. *PM* poor metaboliser, *PCI* percutaneous coronary intervention

**Fig. 2:**
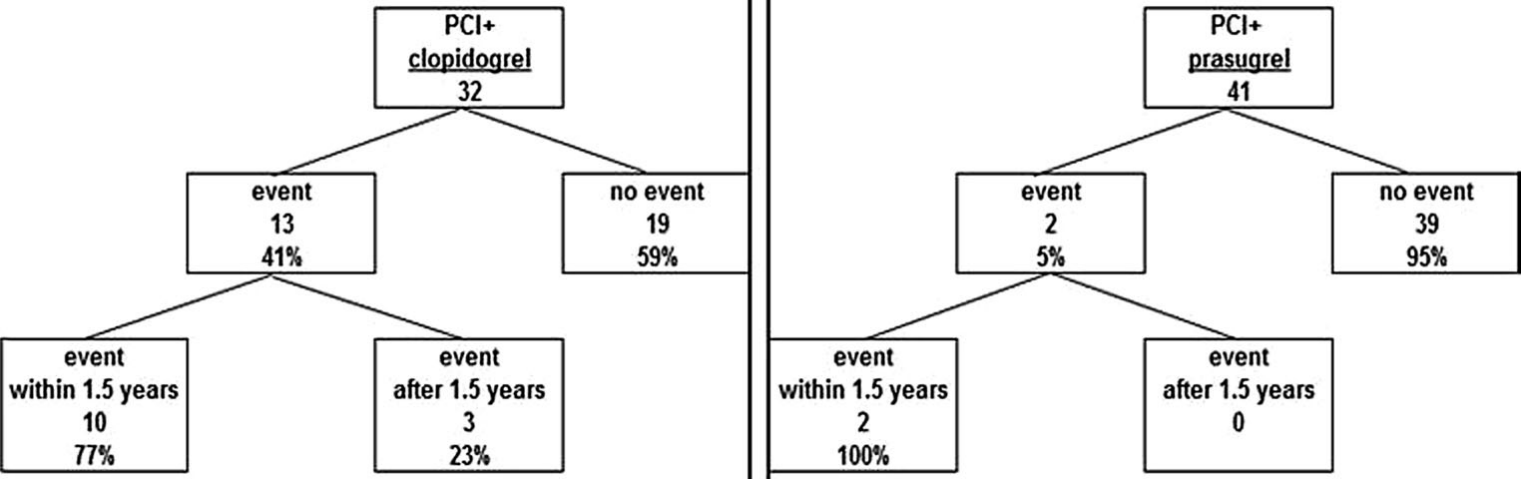
Cardiovascular events in the clopidogrel and prasugrel CYP2C19 poor metaboliser group. An event is defined as death, MI, stent thrombosis or angina pectoris with additional intervention in the same coronary artery. The difference in number of events between the clopidogrel and prasugrel group was found to be significant: *p* < 0.001 for all events and *p* = 0.003 for events within 1.5 years of PCI

Although all patients were scheduled for PCI, only 71 poor metabolisers (CYP2C19*2 homozygote) (80 %) underwent PCI with stent placement. These 71 poor metabolisers were subdivided based on the antiplatelet drug used. Initially, 32 poor metaboliser patients were treated with clopidogrel, 35 with prasugrel, 2 with ticagrelor and for 2 patients the drug used could not be confirmed (Fig. 1). For 7 patients, 6 using clopidogrel and 1 using prasugrel, medication was changed after an additional PCI. Therefore, these patients are part of more than one group. In total, 32 poor metabolisers were enrolled in the clopidogrel group and 41 poor metabolisers in the prasugrel group (Fig. 2). In the clopidogrel group, 19 patients did not have an event, 10 patients had an event within 1.5 years of PCI and for 3 patients this was after more than 1.5 years. In the prasugrel group, 39 patients did not have an event and 2 patients had event within 1.5 years of PCI. No significant difference was observed in the baseline characteristics of the two groups (Table 1). However, the difference in number of events between the clopidogrel and prasugrel group was found to be significant: *p* < 0.001 for all events and *p* = 0.003 for events within 1.5 years of PCI.

**Table 1:** Baseline characteristics of the CYP2C19 poor metabolisers

		Clopidogrel *n* = 32	Prasugrel *n* = 41	*p*-value
Age, mean (SD), years		65.2 (10.2)	64.2 (11.1)	0.680
Sex, *n* (%)	Male	22 (69)	30 (73)	–
	Female	10 (31)	11 (27)	0.680
Race, *n* (%)	Caucasian	32 (100)	40 (98)	–
	Asian	0 (0)	1 (2)	0.370
Medical history, *n* (%)	Smoker	8 (25)	8 (20)	0.600
	Diabetes mellitus	7 (22)	7 (17)	0.630
	Hypertension	17 (53)	14 (34)	0.110
	Hypercholesterolaemia	9 (28)	16 (39)	0.340
	Previous PCI	14 (44)	17 (41)	0.750
	Previous MI	14 (44)	22 (54)	0.420
	Previous CABG	7 (22)	13(32)	0.350
Pharmacotherapy, *n* (%)	Beta-blocker	23 (72)	34 (83)	0.260
	ACE inhibitor	7 (22)	13 (32)	0.350

*CABG* coronary artery bypass graft, *MI* myocardial infarction, *PCI* percutaneous intervention

All three events that took place more than 1.5 years post-PCI were diagnosed as chest pain due to in-stent stenosis, which is not related to the patient’s CYP2C19 genotype. Of the 12 events that took place within 1.5 years of PCI, 9 are categorised as MACE (stent thrombosis, myocardial infarction, death), 8 in the clopidogrel group and 1 in the prasugrel group (*p* = 0.008) (Table 2). There were 6 cases of stent thrombosis, of which 5 definite and 1 probable. Of the 5 definite stent thromboses, 4 occurred in the clopidogrel group and 1 in the prasugrel group. One patient died of stent thrombosis 492 days after PCI as confirmed by autopsy; however, clopidogrel was no longer prescribed at the time of the event. A myocardial infarction in the coronary artery with the implanted stent 374 days after PCI, while the patient was still using clopidogrel, was defined as probable stent thrombosis. Three people died because of stent thrombosis and one patient died of a probable cardiovascular cause. No major bleeding events were reported for these CYP2C19 poor metabolisers. No events occurred in the ticagrelor group but the number of patients using ticagrelor was too small for comparison and statistical analysis.

**Table 2:** Events in poor metabolisers within 18 months of PCI

Events	Clopidogrel *n* = 32 (days after PCI)	Prasugrel *n* = 41 (days after PCI)	Death	Info
Myocardial infarction	46 days	–	No	–
	412 days	–	No	–
Stent thrombosis^a^	–	2 days	Yes	–
	4 days	–	No	–
	4 days	–	Yes	–
	7 days	–	No	–
	492 days	–	Yes	Autopsy
Stent thrombosis probable^a^	374 days	–	No	–
Angina pectoris^b^	–	451 days	No	–
	70 days	–	No	–
	158 days	–	No	–
Unknown	112 days	–	Yes	–
**Total number of events**	**10**	**2**	**–**	**–**

PCI percutaneous intervention

^a^According to the American Research Consortium Statement

^b^Including an additional intervention in same coronary artery

### Stent thrombosis

Among the MACE, the most evidence for an association with CYP2C19-related clopidogrel resistance is shown for stent thrombosis [6, 8–11]. Previously, the increased percentage of stent thrombosis for subjects carrying at least one CYP2C19*2 reduced-function allele compared with non-carriers were reported [6] and the incidence of stent thrombosis was shown to be cumulative according to the number of CYP2C19*2 alleles [8]. Based on these studies, the number of stent thromboses for the Catharina study population in the event that all patients would have been treated with clopidogrel was predicted. For poor metabolisers, the predicted number of stent thromboses was 4 (Table 3), of which 3 are expected to be CYP2C19-related, which is in agreement with the number of stent thromboses found in the present study.

**Table 3:** Predicted number of stent thromboses

Patient		Stent thrombosis			
CYP2C19 phenotype	Number	Chance^a^	Number	CYP2C19-independent	CYP2C19-related
EM/UM	2239	0.008	18	18	–
IM	932	0.026	24	7	17
PM	89	0.043	4	1	3
**Total**	**3260**	**–**	**46** 1.41 %	**–**	**20** 0.61 %

^a^Based on the percentages of stent thrombosis for subjects carrying at least one CYP2C19*2 allele compared with non-carriers (2.7 % vs. 0.8, respectively) as described by Mega and colleagues [6], and the cumulative incidence of stent thrombosis according to the number of CYP2C19*2 alleles [8]. In case of CYP2C19 independence it is assumed that the chances for poor metabolisers (*PM*) and intermediate metabolisers (*IM*) are identical to that of extensive metabolisers (*EM*). The total number of stent thrombosis minus the CYP2C19-independent stent thrombosis is predicted to be the number of CYP2C19-related stent thrombosis

As our results for poor metabolisers are in agreement with the literature, the same is expected to be the case for intermediate and extensive metabolisers. The chance to develop stent thrombosis for intermediate metabolisers is considerably lower than for poor metabolisers (2.6 % vs. 4.3 %, respectively), but still higher than for extensive metaboliser or ultra-rapid metaboliser patients (0.8 %) (Table 3). Because of the large group of intermediate metabolisers this is predicted to result in 24 additional cases of stent thrombosis of which 17 are CYP2C19-related (Table 3).

### Cost-effectiveness

In addition to clopidogrel, prasugrel and ticagrelor can be used to reduce the rate of thrombotic cardiovascular events for patients who undergo PCI [14–16] and are even recommended for most patients with acute coronary syndromes (ACS ) [17, 18]. CYP2C19 is involved in the metabolism of prasugrel, but it does not play a crucial role [2, 5]. Ticagrelor is a direct drug and its metabolism is independent of CYP2C19 activity. However, both agents are much more expensive than clopidogrel and in most countries these agents are only reimbursed for ACS.

Based on the latest prices for clopidogrel, prasugrel and ticagrelor, approximately € 25, € 720 and € 866 per year, respectively, according to the National Health Care Institute (Zorginstituut Nederland, https://www.medicijnkosten.nl/), the pharmaceutical expenses for six different therapy options for the Catharina study population were compared (Table 4). Giving prasugrel to all patients would mean a high increase in pharmaceutical expenses of approximately € 2,200,000 compared with clopidogrel. A less expensive alternative is to give prasugrel only to the high-risk poor metabolisers. This increases the pharmaceutical expenses by just € 60,000, but genotyping is required, resulting in an additional € 270,500 (€ 83 per test according to the latest tariffs of the Dutch Healthcare Authority (NZa)). As intermediate metabolisers also have an increased risk for MACE while on clopidogrel therapy [10], both the intermediate and poor metabolisers could be treated with prasugrel, preventing all the CYP2C19-related events (Table 3). This leads to an increase in pharmaceutical expenses of over € 700,000 and additional costs for genotyping. Because of the higher drug price, these numbers are even higher for both the ticagrelor therapy options (Table 4).

**Table 4:** Cost and QALY

		Patient number	Clopidogrel	Prasugrel	Prasugrel PM	Prasugrel PM + IM	Ticagrelor PM + IM	Ticagrelor
**CYP2C19 phenotype**	EM or UM	2239	€ 55,975	€ 1,612,080	€ 55,975	€ 55,975	€ 55,975	€ 1,938,974
	IM	932	€ 23,300	€ 671,040	€ 23,300	€ 671,040	€ 807,112	€ 807,112
	PM	89	€ 2225	€ 64,080	€ 64,080	€ 64,080	€ 77,074	€ 77,074
**Total pharmaceutical costs**			**€ 81,500**	**€ 2,347,200**	**€ 143,355**	**€ 791,095**	**€ 940,161**	**€ 2,823,160**
Difference PC^a^				€ 2,265,700	€ 61,855	€ 709,595	€ 858,661	€ 2,741,660
Costs genotyping (€ 83/test)				€ 0	€ 270,580	€ 270,580	€ 270,580	€ 0
**Extra costs** ^a^				**€ 2,265,700**	**€ 332,435**	**€ 980,175**	**€ 1,129,241**	**€ 2,741,660**
Incremental QALY^b^				0.018	nd	0.033	0.058	0.105
Gained QALYs^c^				58.68	33.95^d^	107.58	189.08	342.3
**Extra cost/gained QALY**				**€ 38,611**	**€ 9792**	**€ 9111**	**€ 5972**	**€ 8010**

Clopidogrel: € 25/year, prasugrel: € 720/year, ticagrelor: € 855/year

*PM* poor metaboliser, *IM* intermediate metaboliser, *EM* extensive metaboliser, *UM* ultra-rapid metaboliser, *nd* not determined, *PC* pharmaceutical costs, *QALY* quality-adjusted life year, *ST* stent thrombosis

^a^Compared with generic clopidogrel

^b^According to Kazi and colleagues [12]

^c^Gained QALY = incremental QALY × number of patients of study population (*n* = 3260)

^d^This option was not determined by Kazi and colleagues; therefore, the gained QALYs were calculated based on the 8 cases of MACE reported in Table 2 and the estimation that patients aged 65 years having PCI for ACS and being treated with clopidogrel and aspirin for 12 months are projected to have a life expectancy of 9.428 QALYs over their lifetimes [12]. Three patients could have been prevented from dying, which equals 28.3 QALYs. For the 5 patients who did survive stent thrombosis or myocardial infarction a 12 % permanent quality-of-life decrement is assumed [12], which equals 5.66 QALYs. Together, it is estimated that 33.95 QALYs could have been gained

Recently, the results of a cost-effectiveness study of genotype-guided dual antiplatelet therapies for ACS in the United States were presented [12]. It was concluded that treatment with prasugrel or ticagrelor for all patients was relatively expensive, but both genotype-guided therapy with prasugrel or ticagrelor may be an economically reasonable alternative for clopidogrel in dual antiplatelet therapy. The incremental quality-adjusted life years (QALYs) as presented by Kazi and colleagues [12] were used to calculate the gained QALYs for the different therapy options presented above. As expected, the costs per gained QALY for prasugrel for all patients is high, € 38,611 per gained QALY (Table 3). But, for genotype-guided prasugrel and genotype-guided ticagrelor, these costs per gained QALY are much lower, € 9111 and € 5972, respectively. In addition, the gained QALY for ticagrelor for all patients is also low at € 8010 per gained QALY. This difference in incremental cost-effectiveness ratio between prasugrel and ticagrelor was previously explained by the increase of fatal bleeding when using prasugrel [12, 14, 15]. The costs per gained QALY for prasugrel for poor metaboliser patients only, as routinely applied in Catharina Hospital, were estimated to be € 9792.

## Discussion

In this study, based on real-world observations among 3260 elective PCI patients, we found an increased risk for cardiovascular events in CYP2C19 poor metabolisers treated with clopidogrel compared with prasugrel. These results support that CYP2C19 poor metaboliser patients are considered to be at high risk for recurrent events if treated with clopidogrel, as previously described [6, 8–11].

In the Netherlands, the mean ‘willingness to pay’ is estimated to be about € 65,000 per QALY based on the Dutch EQ-5D tariffs and visual analogue scale valuations [19, 20]. Our analysis shows that prasugrel for poor metaboliser patients only, as routinely applied in the Catharina Hospital, could be cost-effective. In addition, it is implementable in daily clinical practice, with relatively low additional costs, and, in the Netherlands, prasugrel in combination with acetylsalicylic acid is approved and reimbursed when prescribed after stent placement, also in case of non-ACS (https://www.medicijnkosten.nl/).

In addition to the high-risk CYP2C19 poor metaboliser patients, also the intermediate metabolisers are at increased risk for MACE [10]. Following the USA [12], our cost-effectiveness analysis shows that also in the Netherlands, the genotype-guided prasugrel and genotype-guided ticagrelor therapy for both CYP2C19 poor metaboliser and intermediate metaboliser patients, and even ticagrelor for all patients could be economically reasonable alternatives for clopidogrel therapy. These results could help in political discussions and discussions with health insurance companies, which may result in changing daily clinical practice to genotype-guided antiplatelet therapy in future.

### Limitations

Although being in line with the literature [6, 8], the number of events is too small to draw any definite conclusions, emphasising the need for further studies to confirm the present results. Baseline characteristics were comparable between the clopidogrel and prasugrel group, but the observational design of the study cannot fully correct for other sources of bias that may have influenced the results. The switch from clopidogrel to prasugrel took place within 1 to 5 days of PCI; however, it is unclear if more events could have been prevented if prasugrel had been given to the poor metabolisers on the day of PCI. Also, it cannot be excluded that the use of ‘second-generation stents’ could have influenced the results [21].

## Conclusion

This study provides evidence that for CYP2C19-related poor metabolisers prasugrel may be more effective than clopidogrel to prevent major adverse cardiovascular events after PCI and this approach could be cost-effective.
